# Case Department

**Published:** 1887-10

**Authors:** 


### CASE DEPARTMENT.

I. Dentigerous Growths.—On May 1st I was called to see a sorrel mare, four years old, that was supposed to have big head (*Osteo Porosis*).

On examination I found the bones over the region of the superior maxillary sinus considerably distended, dull sound on percussion, discharge from the left nostril, breathing on that side considerably affected, gradually losing flesh, although she had an inordinate appetite. An examination of the mouth showed no disease of the molar teeth. I had no hesitation in diagnosing it a disease of the facial sinus, but of what character I could not say. I recommended trephining and removing the foreign substance, to which the owner consented.

I threw and secured the patient. After removing a circular portion of skin and the areolar tissue from over the lower third of the enlargement, and trephined into what was once the superior maxillary sinus, I was astonished to find the place filled with a substance resembling petrified worms, but on closer examination they proved to be teeth. They were enclosed in, and attached to a special membrane, which was vascular and slightly sensitive, and appeared to have the power of producing them.

They were not very firmly attached to the membrane, as I removed the majority of them with my fingers, the rest with a Wolf tooth forceps, as it was the only forceps I had with me. After removing the teeth, I removed the membrane, which was also completely filled with the deposits, being about the size of a millet seed or smaller.

The largest tooth removed was about the size of one's thumb, and an inch and a half long, Their outer structure was very porous and composed of lime; the inner structure was more dense and solid. The points (crown) of several of them were covered with a hard substance resembling enamel. This may appear incredible to some of your readers, but if they had to remove them as I had, they would be convinced of its reality before they were through. On actual count, after the operation, there were 400 teeth, not counting the smaller ones attached to the membrane.

The after treatment consisted in dressing the wound every day with carbolic lotion 1-40, which was still weakened to 1-60. The wound healed up nicely, and the enlargement on the face is gradually disappearing.

A. H. Logan, V.S.

Belfontaine, Ohio.

II. Nasal Polypus.—On the 11th of April of this year a bay mare, aged 8, was brought to my infirmary for examination.

The principal symptom presented was inability to move fast. A distance of twenty or thirty yards was all she could do without danger of going down, apparently from deficient power of respiration.

On examination I found the right nostril closed so that no air passed through, the left also being occluded to a great extent.

The following day I had the animal cast. I then trephined, and found on the right side that the chamber was completely filled with what appeared to be a mass of white fibrous tissue, broken down in places into what resembled pus. By enlarging the opening downwards from the frontal bone for a length of three inches, I found that the growth had not only filled the cavity on the right side, but had so pressed upon the septum nasi as to deflect it greatly over towards the left, thus partly filling up the left chamber.

Evidently, if the right chamber could be cleared, the septum would regain the normal position, to some extent at least, the left chamber being thus enlarged.

I did not wish, however, to attempt a cutting operation in this case, and that for several reasons. I therefore determined to attempt the removal of the neoplasm by medicinal means. I first tried sulphate of copper, but this had no effect. I then combined arsenious acid, one part, with pulverized cupri-sulphas, two parts, and applied this mixture freely.

On examining the tumor the second day after, several portions could be removed. Continuing this treatment, applying the mixture every second day, at the end of sixteen days the nasal chamber was apparently cleared of the growth, it having all sloughed away. Still, however, the hard breathing continued. On the eighteenth (18th) day of treatment I was able to see back into the pharynx, through the chamber. I saw that there was some foreign body lying in the posterior nares. Not knowing what I had got to deal with, I seized the object with a long wire hook, and firmly drew it forward. Gentle yet firm traction was required, when ultimately a tumor dropped into the chamber from which it was easily removed, as its point of attachment to the posterior part of the septum had been torn through. No sooner had this mass been removed than the breathing of the animal was completely relieved.

The polypus measured six inches in length, four inches in circumference, and appeared to be formed of white fibrous tissue. I have preserved the specimen. The cause of the hard breathing was now evident. It had not been wholly due to occlusion of the nasal chambers. The polypus, protruding into the pharynx, and pressing on the epiglottis, had principally produced this condition. Further treatment was simple, and consisted of the use of astringent and healing remedies. Recovery was rapid, the wounds soon healed, and the mare is now doing her usual work every day.

Seville, Ohio. J. H. MILLER, V. S.

III. SPINAL MENINGITIS IN HORSES.—On Wednesday afternoon at 1 P. M., August 24th, I was called to see a horse apparently suffering from laminitis ; the history I obtained from the owner being as follows: On the morning of the same day the animal left the stable in Elizabeth street attached to a heavy cracker wagon on its usual route to Harlem. While the driver was unloading, a heavy rain set in and the horse became thoroughly drenched. After the shower had subsided he was taken back to the stable at about 10.30 A. M., feeling as well as ever, only a little exhausted from the drive. At noon, just as the groom was about to feed it, he noticed that the animal acted as if nailed to the floor, and arrived at the conclusion that the horse was "foundered," as he told the owner, who immediately telephoned me.

Two hours later I arrived at the stable and after a careful examination noted the following symptoms: Membranes congested ; pulse very weak ; temperature normal ; prolonged inspiration; shortened expiration ; ears and extremities cold and a general rigidity of all the muscles. After frequent attempts to move the animal I found it to be impossible, as he evinced intense pain upon every effort.

I admit that I was at a loss as to the nature of the trouble and determined to treat the symptoms. I administered stimulants and anodynes, but the stimulants seemed to have no effect, as I noticed the pulse becoming weaker and weaker. At about 3.30 o'clock the animal began to stagger and showed signs of paralysis ; slings were immediately procured and put in use, but as soon as they were put on he threw his entire weight in them with the fore feet entirely off the ground. I saw they were of no use and that he was suffering more in that condition, and ordered him to be lowered to the floor and the slings removed. I then noticed a complete loss of power in the hind extremities and that he appeared to be choking. He made one or two efforts to eat some oats which were lying on the stable floor but at any attempt to swallow would groan and look to his flank. Hyperdermic injections of strychnia were used, more stimulants were administered, and at 4.30 he died in apparent agony ; cause, failure of the heart's action.

At 7:30 A. M. on the next morning, Thursday, I was again called to the same stable and there found another case almost similar. Before slings could be used the animal was dead.

On the same day, in the same stable, a third case occurred exactly as the first. A plan of treatment was adopted as indicated by the symptoms. Five hours after, the horse died.

On Friday of the same week I was again called to the same place but almost too late to administer any remedies as the animal died just five minutes after my arrival.

On Sunday I was again sent for and informed that another horse was down and seemed to act similar to the others.

Other Veterinary surgeons were called in consultation and arrived at the conclusion, as I had, that the animal was suffering from spinal meningitis. My plan of treatment was laid out to them, which was approved, and we all arrived at the conclusion that the disease was necessarily fatal. The fifth animal died at 4 P. M. on the same day.

During the first three days of the days of the disease, six more cases in the upper part of the city came under my charge but all with the same result. The only difference in the symptoms being the elevation of the temperature, ranging from 101½° to 106½°. The actual cautery over the spine was applied in some cases, as was also physicing. Up to September 1st, twenty-three cases, all in different parts of the city, have been under my care, and about as many more I have heard of through others.

I have read of the disease throughout New Jersey taking the form of cerebro spinal meningitis, but in no instance have I noticed any brain affection.

I will state here that after the first horse died the owner had the stalls thoroughly disinfected, he believing the disease to be contagious. As it has appeared in isolated cases I am inclined to think it infectious rather than contagious.

MARK L. FREY, D. V. S.
New York City.

